# Metabolic Signature of Electrosurgical Liver Dissection

**DOI:** 10.1371/journal.pone.0072022

**Published:** 2013-09-13

**Authors:** Witigo von Schönfels, Oliver von Kampen, Eleonora Patsenker, Felix Stickel, Bodo Schniewind, Sebastian Hinz, Markus Ahrens, Katharina Balschun, Jan-Hendrik Egberts, Klaus Richter, Andreas Landrock, Bence Sipos, Olga Will, Patrizia Huebbe, Stefan Schreiber, Michael Nothnagel, Christoph Röcken, Gerald Rimbach, Thomas Becker, Jochen Hampe, Clemens Schafmayer

**Affiliations:** 1 Department of General and Thoracic Surgery, University Hospital Schleswig-Holstein, Kiel, Germany; 2 Department of Internal Medicine I, University Hospital Schleswig-Holstein, Kiel, Germany; 3 Institute of Clinical Pharmacology and Visceral Research, University of Bern, Bern, Switzerland; 4 Institute of Pathology, University Hospital Schleswig-Holstein, Kiel, Germany; 5 GWT-TUD GmbH, Dresden, Germany; 6 Institute of Pathology, University Hospital Tübingen, Tübingen, Germany; 7 Molecular Imaging North Competence Center, Christian-Albrechts-University Kiel, Kiel, Germany; 8 Institute of Human Nutrition and Food Science, Christian-Albrechts University Kiel; Kiel, Germany; 9 Institute of Medical Statistics and Informatics, University Hospital Schleswig-Holstein, Kiel, Germany; University of Navarra School of Medicine and Center for Applied Medical Research (CIMA), Spain

## Abstract

**Background and Aims:**

High frequency electrosurgery has a key role in the broadening application of liver surgery. Its molecular signature, *i.e.* the metabolites evolving from electrocauterization which may inhibit hepatic wound healing, have not been systematically studied.

**Methods:**

Human liver samples were thus obtained during surgery before and after electrosurgical dissection and subjected to a two-stage metabolomic screening experiment (discovery sample: N = 18, replication sample: N = 20) using gas chromatography/mass spectrometry.

**Results:**

In a set of 208 chemically defined metabolites, electrosurgical dissection lead to a distinct metabolic signature resulting in a separation in the first two dimensions of a principal components analysis. Six metabolites including glycolic acid, azelaic acid, 2-n-pentylfuran, dihydroactinidiolide, 2-butenal and n-pentanal were consistently increased after electrosurgery meeting the discovery (p<2.0×10^−4^) and the replication thresholds (p<3.5×10^−3^). Azelaic acid, a lipid peroxidation product from the fragmentation of abundant sn-2 linoleoyl residues, was most abundant and increased 8.1-fold after electrosurgical liver dissection (p_replication_ = 1.6×10^−4^). The corresponding phospholipid hexadecyl azelaoyl glycerophosphocholine inhibited wound healing and tissue remodelling in scratch- and proliferation assays of hepatic stellate cells and cholangiocytes, and caused apoptosis dose-dependently *in vitro*, which may explain in part the tissue damage due to electrosurgery.

**Conclusion:**

Hepatic electrosurgery generates a metabolic signature with characteristic lipid peroxidation products. Among these, azelaic acid shows a dose-dependent toxicity in liver cells and inhibits wound healing. These observations potentially pave the way for pharmacological intervention prior liver surgery to modify the metabolic response and prevent postoperative complications.

## Introduction

The indications and use of liver surgery have increased substantially over the last decade. Electrosurgical (ES) dissection represents an indispensible tool for blood-sparing liver surgery which has allowed for its broad application [1]–[3]. On the other hand, liver surgery remains a high risk procedure associated with complications such as postoperative haemorrhage, biliary leakage, impaired postoperative liver function, and even liver failure [4], [5]. Although ES has markedly reduced these complications compared to older techniques, significant complications still occur in more than 50% of patients and mortality is estimated to be over 7% for extended resections [6]–[8]. There is considerable evidence from studies performed in the 1970ies and 80ies showing that mono- or bipolar ES adversely affects wound healing in general [3]. ES preparation induces significant thermic and oxidative damage to the liver. Clinically, this surgical trauma is associated with a typical decline in liver function during the immediate postoperative period and clearly affects patient survival [6], [7] resulting from a loss of liver parenchymal reserve in addition to thermal injury to the remaining parenchyma. Thus, measures to minimize electro surgery-derived tissue trauma could potentially improve postoperative outcomes. To develop such interventions, a better knowledge of the toxic metabolites evolving from ES, and their molecular effects on liver cell regeneration is required.

The analysis of the metabolic state of cells or tissues has recently been facilitated by the development of novel techniques which allow for an increasing number of compounds to be quantified from biological material that has collectively been termed metabolomics. These techniques encompass a heterogeneous set of methods including gas chromatography/mass spectrometry (GC/MS), high pressure liquid chromatography/mass spectrometry (LC/MS) and nuclear magnetic resonance (NMR). Although these emerging techniques are limited both in the number of identifiable metabolites and the applicability to complex biological matrices, they are increasingly contributing to an improved understanding of disease pathophysiology and biomarker discovery.

Here, we employ a GC/MS discovery platform to investigate the metabolic signature of ES in human liver in a two stage design and identify a characteristic lipid peroxidation profile. The most abundant metabolite, namely azelaic acid, which also exhibits the largest degree of change among the replicated metabolites increased after ES liver dissection is investigated further using *in vitro* assays that demonstrate potent anti-proliferative and anti-wound healing properties. Thus, the findings presented contribute to a better understanding of the molecular signature of ES dissection and may provide the biological background to create perioperative pharmacological interventions in the future.

## Patients and Methods

### Patient samples

Liver samples were obtained during open or laparoscopic surgery in patients in whom an intraoperative liver biopsy was indicated on clinical grounds such as during scheduled liver resection, exclusion of liver malignancy during mayor oncological abdominal surgery or assessment of liver histology during bariatric surgery. Electrocautery was used during laparotomy as needed clinically for hemostasis during abdominal wall dissection. A sharp liver biopsy comprising approximately 50 mg of liver tissue in addition to the tissue needed for histopathological analysis was performed before any ES was used on the liver. Hemostasis on the biopsy site was achieved using ES coagulation using the forced coagulation setting of the ES unit (60W maximum power setting, Valleylab Force 2™, Force FX™ or Force Triade™, Boulder, CO, USA). A further sharp biopsy including the coagulated liver surface was then obtained. Liver dissection was then continued as clinically indicated. All patients provided written, informed consent on the day before surgery. The study protocol was approved by the institutional review board (“Ethikkommission der Medizinischen Fakultät der Universität Kiel”, D425/07, A111/99) before the commencement of the study. Samples were placed in screw-cap cryotubes and immediately frozen in liquid nitrogen in the operating room ensuring an *ex-vivo* time of less than one minute in all cases. Samples before and after ES were stored in adjacent tubes in liquid nitrogen racks until processed. Discovery and replication samples were divided by recruitment date and measured in August 2010 and June 2011, respectively.

### Sample preparation and derivatisation for metabolite analysis

A one step extraction/methylation method using trimethylsulphonium hydroxide (TMSH) [9]–[11] as agent for methylation and protection from further metabolic activity in the sample was used as follows: Ten milligrams of frozen tissue were ground with 5 mm stainless steel balls using liquid nitrogen cooling (MM301 Mixer Mill,Retsch®, Haan, Germany) for 30 seconds to a fine powder. For blank samples, all steps were performed identically in parallel to the patient samples, but without tissue. The ground powder was suspended by adding 200 µl methanol (Merck, Darmstadt, Germany) at −20°C and transferred to holding tubes where it remained for up to 15 minutes using gentle agitation on a block cooled to −20°C with dry ice. The fully suspended vial content was then transferred into a tapered 1.1 ml GC vial with screw cap (1.1-STVG, 8-ST15, Thermo Fischer Inc., Waltham, MA, USA) and the solution was immediately supplemented with 40 µl 25% TMSH in methanol (Jenachem™, Jena, Germany) as a methylating agent followed by 15 minutes of incubation at 100°C for derivatisation [9]. To reduce the pressure load on the glass vials and to reduce evaporation, each vial was heated within a 50 ml plastic tube (Falcon™, BD Biosciences, Heidelberg, Germany) containing 100 µl of methanol as an outer vial. After derivatisation, 80 µl of the clear supernatant were transferred into new GC vials. Then, the GC vials were opened and all liquid was allowed to evaporate at room temperature. For preparation of an analytical reference, each 10 mgs of one tissue sample of the same patient before and after electrocautery were combined. The preparation of the phospholipid and triglyceride fractions are described in Methods S1 in File S1.

### Analytical method

A VocIdent-CIMS method combining solid phase micro extraction (SPME on a DVB/Car/PDMS, 57329-U fiber, Sigma-Aldrich, Taufkirchen, Germany) of volatile organic compounds (VOC) and a full evaporation technique [12] with GC CIMS was used. GC separation on a polar column (Rtx Wax, 15 m, 0.25 mm i.d., 0.25 µm film thickness, Restek, Bad Homburg, Germany) carrier and Ion Trap MS detection using internal chemical ionisation (CI) with water as reagent gas [13] (GC 431 and Ion trap MS 220 and MS Workstation V 6.9.3, Varian, Darmstadt, Germany) [14] was used. The opened 1.1 ml–sample vials were inserted into 40 ml EPA vials with screw cap and septum (Restek), capped and heated for at least one hour at 100°C. Analytes were then collected on the SPME fiber for 18 min and subsequently injected for 1 min at 260°C into the GC at a column temperature of 40°C and ramped with 15 degrees/min to 240°C. Helium was used as carrier gas at a constant pressure of 8 psi. Trap temperature was 140°C. This resulted in a GC cycle time of 20 min. For all samples, two consecutive SPME were performed from the same GC vial. Measurement batches were automatically performed over a time period of 8–20 hours and contained each three to eight paired samples before and after electrosurgical resection, two to four empty control vials and two to four mix samples for standardization purposes.

### Identification of the analytes

The mass spectrometry (MS) data (scan range 33–400 m/z) was processed using the MS Workstation Software (Varian V6.9.3) and identification of the analytes was based on retention index and mass spectra according to a compound library of ∼1800 CI mass spectra. In more detail, in mass spectrometry using an ion trap detector based on chemical ionisation with water as a reactant, the analyte M becomes a positive charged ion by transfer of a proton (denoted MH^+^). The soft chemical ionisation causes significantly less fragmentation of the compounds than electron impact ionisation. (Figure S1 in File S1) shows the CI mass spectrum of azelainic acid dimethylester of the reference compound (Chiracon GmbH, Luckenwalde, Germany). The peak area for each compound was calculated using the specific masses and is reported in dimension less “counts”. Peak areas for all metabolites were first normalized on the overall median total peak area of all samples. Secondly, the two extractions of each patient and mix sample were merged retaining the average of the two peak areas for each metabolite. Finally, the ratio of the median area for each metabolite in the mix standards during a measurement batch as compared to its overall mean was calculated and the metabolite counts of the patient samples adjusted accordingly.

### Data handling and quantification of azelaic acid

Data handling, the quantification of azelaic acid, the in vitro assays and statistics are described in Methods S1 in File S1 and Table S2 in File S1.

### Cellular in vitro assays

All cell lines (CFSC-2G, MMNK-1, HepG2) were kept under the standard conditions – DMEM or RPMI supplemented with 10%FCS, 200 IU/mL penicillin, 200 µg/mL streptomycin (Biochrom, Berlin, Germany) in a humidified atmosphere of 95% air with 5% CO_2_ at 37°C. For all the experiments, after reaching the semi-confluent state, cells were starved for 24 h in serum-free conditions and then treated with HAzPC (Cayman Chemical) for the next 24–72 h at 100 nM–10 µM concentrations and repeated at least 2 times if not stated otherwise. Vehicle control treated groups were excluded from the experiments as no effect of solvent (ethanol) was found.

#### Migration assay

Cell migration was assessed using an *in vitro* wound closure assay. After 24 h of starvation, a scratch was generated in a confluent monolayer of cells using 100 µl pipette tip. 5 µM HAzPC was added to the serum-free medium and the initial scratch width was compared to the scratch width after 24 h of incubation with a net micrometer at 3 predetermined points per well.

#### Proliferation assay

Cells were seeded at a density of 2×10^4^ per well in 96-well plates. After 24 h of starvation, the cells were stimulated by 0.2–10% FBS in the presence of absence of 100 nM–10 µM HAzPC for 24 h. BrdU was added during the last 4 h and the amount of BrdU incorporation determined by a colorimetric BrdU cell proliferation enzyme-linked immunoassay (ELISA) according to the manufacturer's instructions (Roche, Basel, Switzerland).

#### TaqMan PCR analysis

RNA was isolated from from 5×10^5^ cells using the RNAeasy kit (Qiagen) according to the manufacturer's recommendations. cDNA was transcribed from 1 µg of RNA obtained using MMLV reverse transcriptase (GibcoBRL) with a random hexanucleotide mix (Roche). TaqMan probe and primer sets (PCαI, αSMA, TGFβ1, MMP-3, TIMP-1, TNFα, IL-6, iNOS, COX-2) are obtained from Applied Biosystems (Rotkreuz, Switzerland) as ready to use kits. For normalization, the housekeeping gene glyceraldehyde-3-phosphate dehydrogenase (GAPDH) was amplified in a parallel reaction.

#### Western Blotting

The proteins were isolated from 5×10^5^ cells, separated by SDS-PAGE and transferred to nitrocellulose membrane. Membranes were blocked and incubated with the primary antibody raised against proteins specified (bax, bcl-2, caspase-3 (total and cleaved), collagen I, actin, fibronectin) overnight (or 48 h) at +4°C and incubated with the corresponding horseradish peroxidase-conjugated secondary antibody. Immunodetected proteins will be visualized using the enhanced chemiluminescence ECL assay kit (Amersham Biosciences, Freiburg, Germany).

#### Cell viability

5×10^5^ cells per well in 12-well plates were starved for 24 h and stimulated then by 100 nM–10 µM HAzPC for 24 h. Trypan blue stained (non-viable) cells were counted and the values are expressed as x-fold change to control.

## Results

The experiment was conducted in a two-stage design using a total of 36 paired liver samples before and after ES. In the discovery series, between two and three independent paired liver samples were obtained from six individuals, extracted and subjected to metabolic screening using GC/MS. The replication panel consisted of 20 paired samples obtained from 20 patients. An overview of the descriptive clinical, histological and demographic data for the samples in both series is provided in Table 1.

**Table 1:** Overview of the patients used for metabolic screening of the effects of ES on human liver.

		screening	replication
Number of paired samples		16	20
Number of patients		6	20
Median age		70 [59–77]	67 [29–86]
Median BMI		26 [22–32]	25 [20–57]
Sex (% female)		100%	80%
Histology	Fibrosis	0 [0–0]	0 [0–2]
	Fat (%)	8 [0–30]	3 [0–80]
	Inflammation	0 [0–0]	0 [0–3]

In all cases, paired samples with and without electrosurgical coagulation were obtained. For the discovery stage, two to three independent pairs of samples from each patient and in the replication stage exactly one pair of samples were obtained. The range of the numeric parameters is given in square brackets.

### Metabolic signature of electrodissection (ES) in human liver surgery

**Figure 1:**
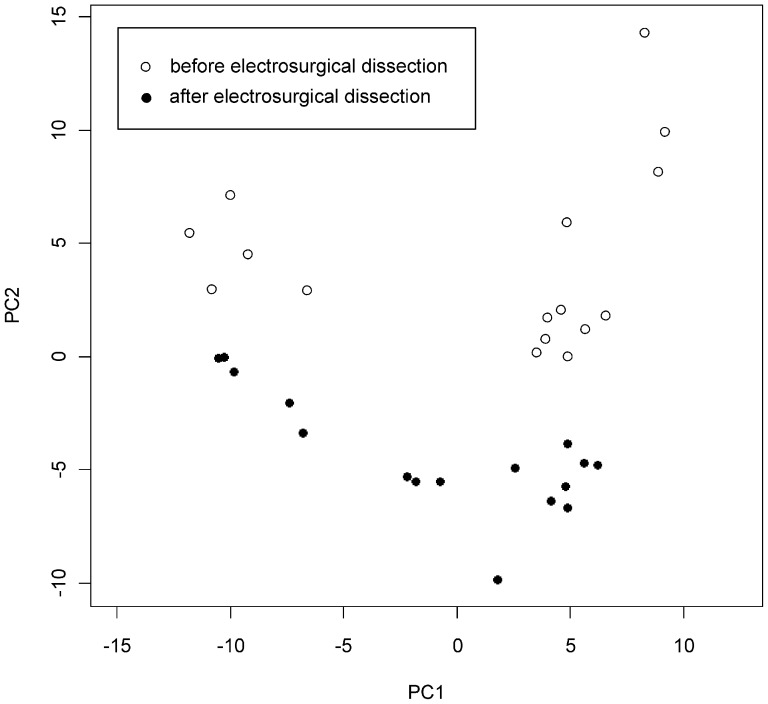
Principal components analysis of the samples of the discovery stage. The first two principal components are plotted. Samples before and after ES are marked with open and filled circles, respectively.

**Figure 2:**
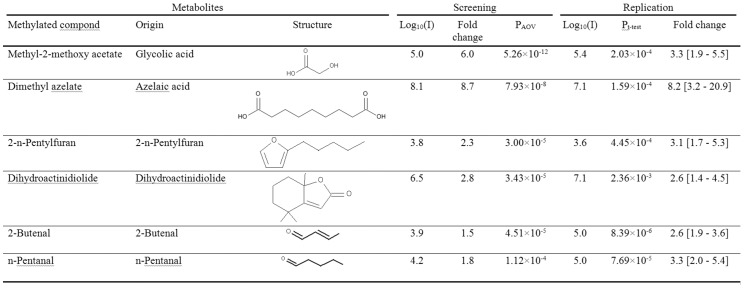
Metabolites identified as consistently increased after ES in liver samples. The results of the screening stage are noted with the decadic logarithm of the peak area in the GC/MS denoted as Log10(I), the fold increase after ES and the p-value obtained from the analysis of variance including the patient as covariate denoted as PAOV. For the replication series, results of the paired t-test are denoted as Pt-test. Nominal p-values are given for all analyses.

**Figure 3:**
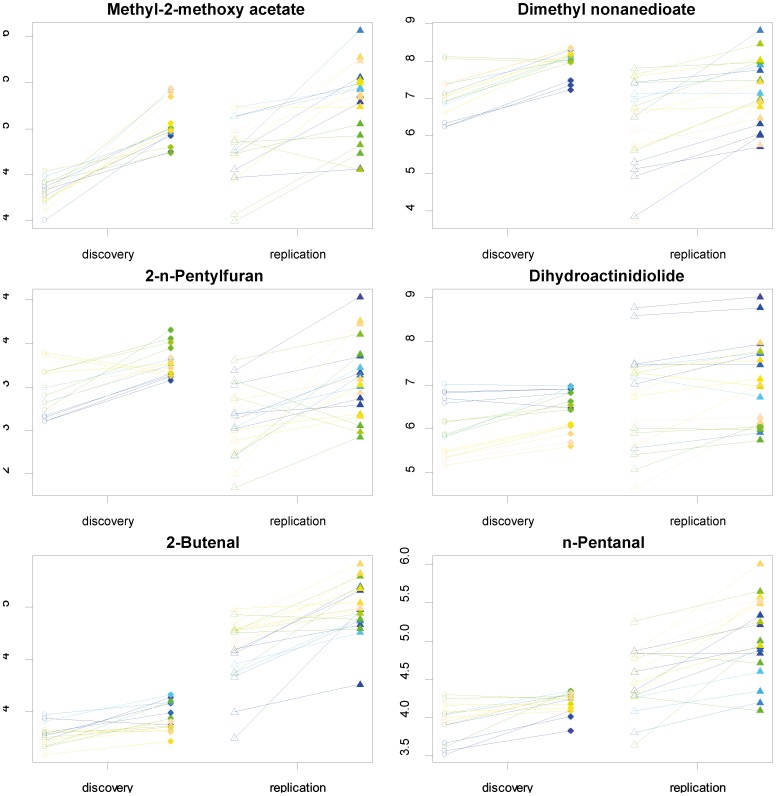
Log10 concentrations for each of the six replicated metabolites before (denoted with open symbols) and after ES (denoted with filled symbols). The paired samples are each connected with a line and pairs are coloured differently for better visualization.

Metabolites underwent filtering and quality control as described in Methods S1 in File S1. As an exploratory step to evaluate the global metabolite pattern with regards to the effects of ES, a principal components analysis was performed in the discovery sample set. A separation of the samples before and after ES was seen in the first two principal components (**Fig. 1**). In order to define specific metabolites that are increased after ES liver dissection, the log 10 transformed peak areas for each metabolite were compared before and after ES in the discovery set. The metabolite-specific significances for increased compounds were ranked by the p-value in the analysis of variance using the patient identifier as factor. A Bonferroni corrected threshold of p<2.0×10^−4^ was adopted for statistical significance, corresponding to 208 tests. A total of 14 metabolites passed this threshold and were then evaluated in the second stage, adopting a Bonferroni-corrected threshold of 3.5×10^−3^ for the paired t-test. The details of the two-stage analysis for all 208 compounds are provided in Table S1 in File S1. Six metabolites, namely methyl-2-methoxy acetate, dimethyl azelate, 2-n-pentylfuran, dihydroactinidiolide, 2-butenal and n-pentanal could be replicated and show thus a consistent increase after ES in human liver (Fig. 2, Fig. 3). In addition, a non-parametric test (Mann-Whitney U-test) was performed over all metabolites and confirmed the replication findings (Table S1 in File S1). In order to allow extraction of metabolites into the gas phase for GC, the samples underwent derivatisation with TMSH before analysis and these methylated derivates are listed in both Figure 2 and Table S1 in File S1. The respective unmethylated substances of origin for the replicated metabolites are noted with their chemical structure in Figure 2 and correspond for the validated metabolite set to glycolic acid, azelaic acid, 2-n-pentylfuran, dihydroactinidiolide, 2-butenal and n-pentanal. The chemical properties of these substances are noted in more detail in the Discussion.

### Quantification of the concentration of azelaic acid

For further in-depth analysis, we selected azelaic acid as the metabolite that showed both the highest relative changed in both the discovery (8.7-fold) and replication data sets (8.2-fold 95% CI: 3.2–20.9) and had the highest median intensity count in samples post ES (Fig. 2). Using pure synthetic dimethyl azelate – the dimethyl ester of azealic acid generated by derivatisation using TMSH – the GC/MS signature and thus the chemical assignment of this substance was confirmed. Using a synthezised standard compound (azalaic acid methyl ester), a response factor of 1×10^7^ counts per ng was estimated (Figure S2 in File S1). Thus, the measurement range corresponds to an approximate amount of 1–10 ng of dimethyl azelate in the vial. Given further, that 5 mg (corresponding to an approximate tissue volume of 5 µl) of specimen were processed and a molecular weight of 216 g/mol for dimethyl azelate, the tissue concentration ranged between 1 µM to 10 µM across the liver specimens. Because azelaic acid was identified as the metabolite found consistently and significantly increased in both investigated sets of samples, this substance is likely a result of ES dissection with its highest concentration on the plane of resection. We thus estimate that the local tissue concentration the site of thermal injury will be approximately of 5 to 10 fold higher than the amount measured across the full liver specimen. Thus, the range of tissue concentration would correspond to a range from 100 to 5 µM concentrations at the site of the biological effect.

Further, in order to obtain an estimate of the distribution of azealic acid residues in the liver after ES dissection, four paired ES treated samples from a patient with sufficient patient material were extracted without derivatisation by TMSH. A median peak area of 3.1×10^5^ counts for dimethyl azelate was obtained which was two to three orders of magnitude lower than in the samples after TMSH derivatisation that exhibited median peak area of 10^7^ to 10^8^ counts. Given, that the peak area in patient vials was between 14495-fold (discovery) and 2336-fold (replication) fold higher as in empty control vials (Table S1 in File S1), these peak areas are in the range of the control vial intensities indicating, that free azealic acid is present in a very low concentration and thus does not account for the observed ES effect. Secondly, the median abundance of dimethyl azelate in the TMSH-treated triglyceride (Log_10_(I) = 7.2) and phospholipid fractions (Log_10_(I) = 7.1) from these samples were similar, indicating that the quantitatively dominant amount of azealic acid was bound in triglycerides and phospholipids in human liver.

Azealic acid is a known lipid peroxidation product of C18 monounsaturated fatty acids [15], [16], nameley oleic acid (18∶1 cis-9) and elaidic acid (18∶1 trans-9). Oleic acid is extremely abundant in the analysed liver samples with Log_10_(I) intensities >10, and is thus beyond the linear range of the detector and was thus not quantitatively analysed. The fatty acid methyl ester (generated by methylation by TMSH: FAME 18∶1 trans-9) of elaidic acid has Log_10_(I) intensities greater 8 in both series is thus the most abundant quantified metabolite (Table S1 in File S1). As expected, the presence and abundance of azealic acid thus corresponds to the presence of the respective precursors.

### Cellular assays

**Figure 4:**
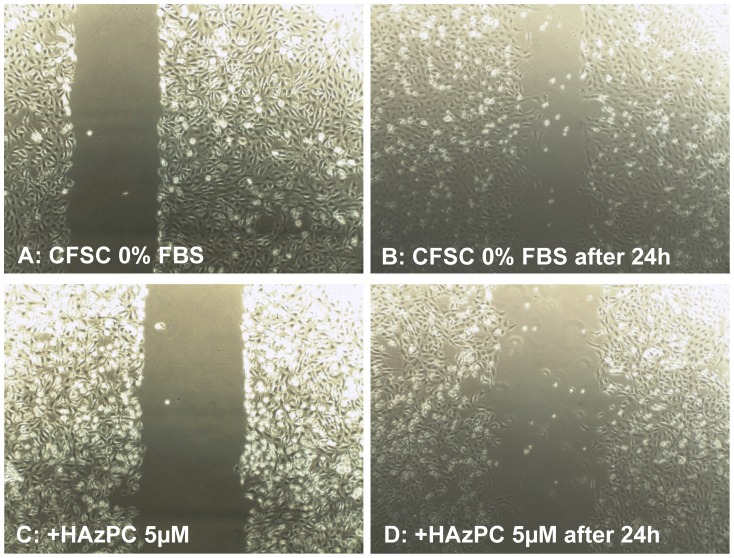
Incubated cells with HAzPC. Incubation of cells with HAzPC at 5 µM in a wound healing scratch assay demonstrated that closure of the scratch occurs more rapidly in the absence of HAzPC (upper panels A and B) than with HAzPC treatment (lower panels C and D).

Azelaic acid is formed by oxidative fragmentation of the 9,10-double bond (14) of the most abundant unsaturated (oleoyl and elaidyl) residues of phospholipids, the majority of which remain esterified to the phosphatidylcholine backbone [15], [16]. Thus, the synthetic phosplipid hexadecyl-azealin phosphadidylcholine (HAzPC) was used to evaluate its potential effects on wound healing in the liver. Specifically, rat HSC (CFSC-2G) and immortalized human bile duct epithelial cells (MMNK-1) were treated with HAzPC at concentrations ranging from 100 nm to 5 µM for 24 h to assess cell proliferation and gene transcription related to remodeling and wound healing. HAzPC inhibited DNA synthesis in both cell lines dose-dependently (Figure S3, Table S3 both in File S1), and modified the expression of genes involved in tissue repair by CFSC-2G cells (Figure S4 in File S1) and, in a similar fashion in MMNK-1 cells (data not shown) at HAzPC concentrations typically evolving during electrocautery. Consistent with these effects, HAzPC at 5 µM retarded the closure of a scratch employed in a wound closure assay compared to untreated control (Figure 4): A significantly impaired closure of the scratch was observed for CFSC-2G with a −7% width reduction as compared to 24% with medium alone after 24 hours. In MMNK-1 cells, similar results were obtained with recorded width reductions of 29% as compared to 57% with 5 µM HAzPC and medium alone after 24 hours, respectively.

**Figure 5:**
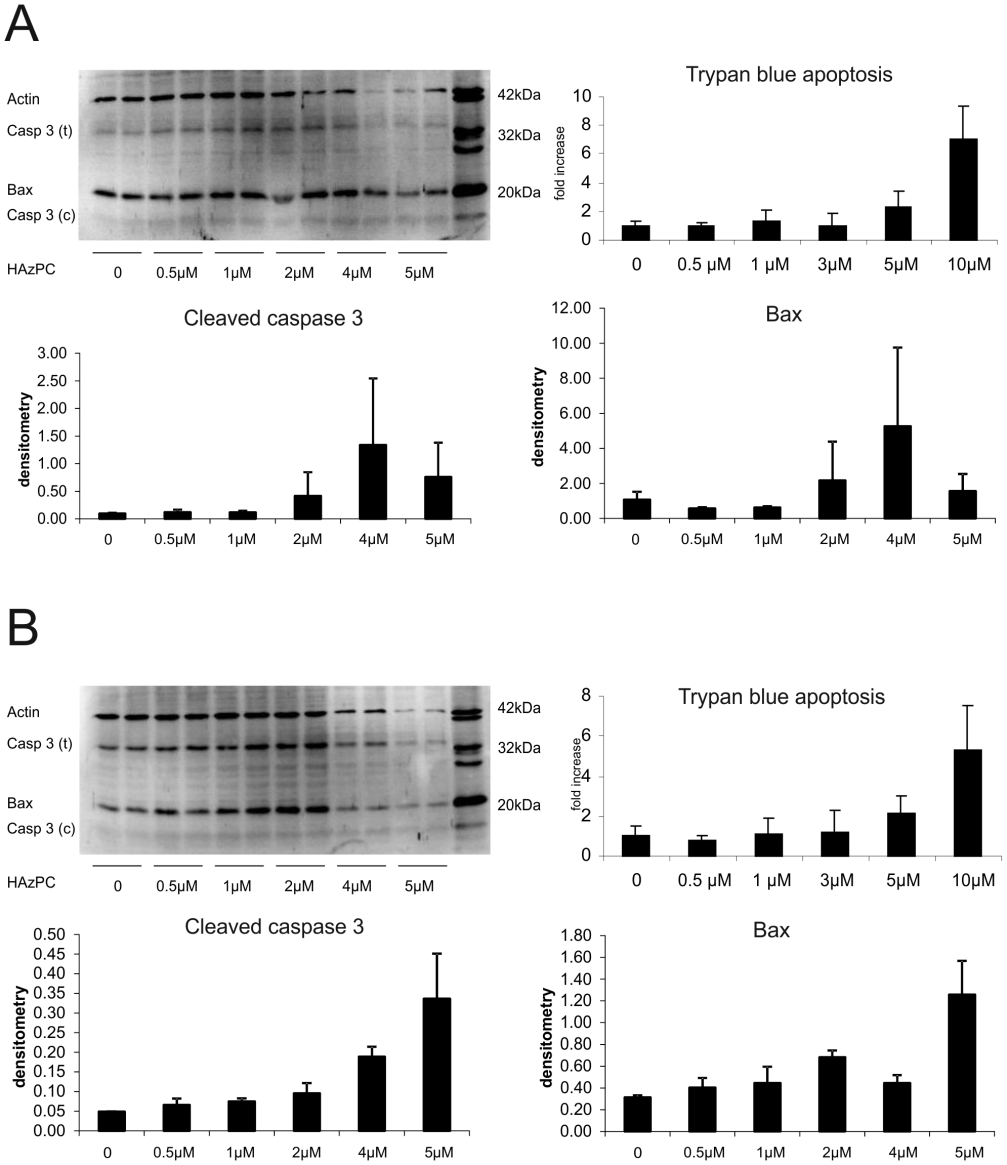
HAzPC dose-dependently increased apoptosis as reflected by an increase of Trypan Blue inclusion in CFSC-2G (A) and MMNK-1 (B) cells. Cell morphology suggesting apoptosis was confirmed by a dose-dependent increase of cleaved caspase and pro-apoptotic protein bax in both cell lines. The decrease of protein expression including that of cleaved caspase 3 (C) and bax at 5mcrmol of HAzPC in Western Blots likely reflects cell demise.

To investigate possible effects of HAzPC on cell viability, trypan Blue stains for cell death and Western Blotting for specific propapoptotic proteins activation were performed. HAzPC induced apoptotic cell death at concentrations starting from 5 µM, via activation of caspase-3 and bax signalling pathways in both CFSC-2G (**Fig. 5A**) and MMNK-1 (**Fig. 5B**) cell lines.

## Discussion

In this report, we provide the first systematic analysis of the metabolic effects of electrosurgical dissection on the liver using a two stage metabolomic screening experiment and define a characteristic signature of lipid peroxidation products. For azealic acid, the metabolite, that shows both the highest relative fold change and at the same time is most abundant among the compounds of the replicated metabolic signature, we demonstrate an inhibition of wound healing and pro-apoptotic effects with the typical tissue concentrations observed after ES *in vivo*.

While global analyses of metabolism are increasingly contributing to an improved understanding of disease pathophysiology and biomarker discovery [17]–[19], the field of metabolomics is still at an early stage. Our study reflects these limitations, for instance in the limited number of metabolites that were analysed. However, this study still provides the first global metabolite analysis of ES liver dissection using the accessible metabolite spectrum of the reported GC/MS method. The application of chemical ionisation as opposed to electron impact ionisation allowed the analysis of a large number of chemically defined metabolites from the complex biological matrix [14]. Owing to the hypothesis-generating nature of the screening experiment and in order to avoid the introduction of contaminants, no antioxidant or chelate forming additives were used and a single step method of extraction/methylation [9]–[11] was used, that minimized the number of sample handling steps. Larger samples sizes and improved technologies will lead to a more complete understanding of the resulting molecular pattern in the future.

As the molecular signature of electrosurgical dissection is as yet a grossly under-researched topic, the evidence for pathway assignment of the reported compounds has been generated in classical organic chemistry [15] or, more recently, in the context of thermic processing of agricultural products or enzymatic lipid oxidation processes. While glycolic acid is a known product of sugar oxidation in general [20] and of ribose oxidation from the ribonucleic acids [21] in particular, all other identified metabolites can be attributed to different lipid oxidation pathways:

Dihydroactinidiolide is a beta-carotene cleavage product produced by stimulated neutrophils *in vitro* [22] and is generated during beta-carotene degradation by hypochlorous acid [23]. The metabolites pentylfuran, butenal and pentanal have been reported in conjunction with the oxidative degradation of linoleic acid. Dry-roasting conditions in food processing were shown to promote the formation of 2-alkylfurans from the corresponding lipid-derived α,β-unsaturated aldehydes [24]. Specifically, the formation 2-pentylfuran from 2-nonenal, an intermediate of lipid peroxidation, was demonstrated [25]. Pentanal and 2-butenal represent further downstream products of the lipid peroxidation of linoleic acid [26], [27].

Azelaic acid is a typical oxidation product from the most abundant unsaturated (oleoyl and elaidyl) lipid residues [15] of triglycerides and phospholipids. The majority of these residues remain esterified to the phosphatidylcholine backbone after oxidation to azealic acid [15], [16]. Here, we demonstrate in cellular assays, that a typical synthetic representative of these phospholipids, namely hexadecyl-azealin phosphadidylcholine (HAzPC) impairs tissue remodelling and wound healing and triggers apoptosis in cellular models for myofibroblasts and cholangiocytes, two hepatic cell types centrally involved in tissue repair. Previous reports in other cellular systems have shown, that the pro-apoptotic properties of HAzPC are mediated through mitochrondrial damage and caspase activation [28], [29]. While future mechanistic studies need to determine the exact molecular links to tissue remodelling and wound healing, the activities of caspases could be pharmacologically blocked and could thus represent potential targets for perioperative interventions in order to minimize the tissue damage triggered by ES. Alternatively, upstream pharmacological interventions using for instance vitamin E as an inhibitor of lipid peroxidation could represent a potential therapeutic angle [30], [31]. Clearly, a broader exploration of lipids containing azealic acid is required in the future.

Liver regeneration is regulated via cell-cell and cell-matrix interactions involving an array of different liver cells [32]–[34]. After liver resection, hepatocyte growth factor (HGF), transforming growth factor (TGF) β1, epidermal growth factor (EGF), tumour necrosis factor (TNF) α/β and interleukins 1β and 6 are among the main growth-mediating factors [35]. Transcription factors including nuclear factor kappa B (NFκB) and STAT3 are recruited to increase transcription of genes that promote liver regeneration. In light of the results of the data presented in this study, the links between these fundamental liver regeneration pathways and the downstream effectors of the described oxidation products would represent an intriguing new field of research.

In summary, given the key role of electrosurgical liver dissection in the treatment of liver tumours, metastases and transplantation and its broadening application both with regards to the extent of resection and the underlying patient co-morbidity, the minimization of tissue and systemic trauma during liver surgery is of paramount importance. This study may serve as a starting point towards a mechanistic understanding of its molecular signature and thus potentially pave the way for pharmacological intervention prior liver surgery to modify the metabolic response and prevent postoperative complications.

## Supporting Information

File S1 **Figure S1, Example of analyte identification using GC/MS with chemical ionisation (CI) using water as reactent gas. Figure S2, Peak areas for 13 extraction steps of a vial. Figure S3, DNA-synthesis of CFSC-2G (panel A) and MMNK-1 cells. Figure S4, Analysis of mRNA expression using real-time PCR in CFSC-2G cells exposed to different concentrations of HAzPC. Methods S1, Additional information concerning the methods. Table S1, List of all analysed compounds in the metabolic screening experiment. Table S2, Quantitation data from multiple extractions of a sample vial spiked with 75 ng azelaic acid dimethylester. Table S3, Rat (r) and human (h) primers and probes used for TaqMan PCR.**
(DOCX)
