# Supplementary material for: Metabolic Signature of Electrosurgical Liver Dissection
Source: PLoS One. 2013 Sep 13;8(9):e72022. doi: 10.1371/journal.pone.0072022 (PMC3772850; doi:10.1371/journal.pone.0072022)
Supplement: File S1 — Figure S1, Example of analyte identification using GC/MS with chemical ionisation (CI) using water as reactent gas. Figure S2, Peak areas for 13 extraction steps of a vial. Figure S3, DNA-synthesis of CFSC-2G (panel A) and MMNK-1 cells. Figure S4, Analysis of mRNA expression using real-time PCR in CFSC-2G cells exposed to different concentrations of HAzPC. Methods S1, Additional information concerning the methods. Table S1, List of all analysed compounds in the metabolic screening experiment. Table S2, Quantitation data from multiple extractions of a sample vial spiked with 75 ng azelaic acid dimethylester. Table S3, Rat (r) and human (h) primers and probes used for TaqMan PCR. (DOCX) [file pone.0072022.s001.docx]

# Supplementary Tables

## Supplementary Table 1 (Table S1)

List of all analysed compounds in the metabolic screening experiment. The compounds are listed with their chemical structure according as identified in the GC/MS, i.e. after derivatisation using TMSH. For each compound, the chemical name, Log10 intensities in samples after ES dissection Log_10_(I), the control score (i.e. the relative intensity in patient samples and mix samples as compared to empty controls – see Results), the logarithmic (base 10) difference between samples before and after ES (Logdiff: positive numbers indicate increased metabolite concentrations after ES) and the nominal p-values in the analysis of variance in the discovery series (P_AOV_) and the p-values from the U-tests and t-tests from the replication series are provided. For the logarithmic difference in the replication series, 95% confidence intervals are given. Metabolites are ranked according to the discovery p-value (P_AOV_) in increasing order. None of the compounds showed a significant deviation from normality in the discovery set (Komolgorov-Smirnov test p≥0.05). Two compounds (FAME C12:0 and FAME C14:0) showed deviations of the log10 differences (Logdiff ) from normality (0.02 and 0.0004, respectively) in the replication set and are marked with an asterisk.

|  | **Discovery** | | | | **Replication** | | | | |
| --- | --- | --- | --- | --- | --- | --- | --- | --- | --- |
| **Metabolite** | **Log10(I)** | **Control score** | **Logdiff** | **P_AOV_** | **Log10(I)** | **Control score** | **P(U-test)** | **P(t-test)** | **Logdiff [95% CI]** |
| Methyl-2-methoxy acetate | 4,99 | 11,7 | 0,78 | 5,26E-12 | 5,37 | 28,5 | 0,00029 | 0,00020 | 0,51 [0,29 - 0,74] |
| Dimethyl nonanedioate (azelate) | 8,12 | 14495,6 | 0,94 | 7,93E-08 | 7,05 | 2336,0 | 2,67E-05 | 0,00016 | 0,91 [0,51 - 1,32] |
| 2-Heptanol | 4,07 | 14,1 | -0,55 | 8,16E-08 | 4,27 | 572,8 | 0,00129 | 0,00280 | 0,58 [0,23 - 0,93] |
| Dimethyl heptanedioate | 6,60 | 8970,2 | 0,61 | 1,40E-07 | 7,03 | 1000,0 | 0,00391 | 0,01676 | 0,60 [0,14 - 1,07] |
| Tetramethyl aspartate | 4,47 | 346,3 | 0,73 | 2,13E-07 | 1,68 | 1000,0 | 0,62500 | 0,33040 | 0,24 [-0,42 - 0,91] |
| 1,6-Dioxa c-C12-7,12-dione | 5,70 | 9565,6 | 1,26 | 2,79E-07 | 0,48 | 1000,0 | 0,06250 | 0,07457 | 0,41 [-0,06 - 0,88] |
| N-Me-2-pyrrolate | 3,89 | 50,8 | -0,26 | 3,54E-07 | 4,83 | 1000,0 | 6,10E-05 | 3,41E-05 | 1,24 [0,79 - 1,70] |
| FAME cis-C15:1n10 | 4,73 | 35,5 | 0,55 | 5,88E-07 | 4,05 | 27,2 | 0,01099 | 0,00778 | 0,90 [0,27 - 1,52] |
| Tetramethylurea | 3,78 | 103,9 | -0,84 | 2,81E-06 | 0,48 | 37,4 | 0,50000 | 0,11799 | 0,99 [-1,37 - 3,35] |
| Acetonitrile | 4,21 | 5,7 | -0,21 | 9,24E-06 | 5,26 | 24,9 | 0,00032 | 0,00028 | 0,34 [0,18 - 0,50] |
| N-Methylisobutyramide | 3,53 | 81,7 | -0,54 | 1,62E-05 | 3,91 | 121,2 | 0,06396 | 0,05613 | 0,32 [-0,01 - 0,65] |
| Me-2-(OHimino)propanoate | 5,33 | 753,5 | 1,11 | 1,66E-05 | 0,48 | 3691,8 | 0,62500 | 0,43616 | 0,27 [-0,70 - 1,25] |
| 2-n-Pentylfuran | 3,75 | 15,7 | 0,36 | 3,00E-05 | 3,56 | 416,2 | 0,00085 | 0,00045 | 0,49 [0,25 - 0,73] |
| Dihydroactinidiolide | 6,51 | 6040,4 | 0,44 | 3,43E-05 | 7,08 | 167864,5 | 0,00169 | 0,00236 | 0,41 [0,17 - 0,66] |
| 3-Hexanone | 3,95 | 8,5 | -0,27 | 4,16E-05 | 4,24 | 30,3 | 0,00129 | 0,00110 | 0,25 [0,11 - 0,38] |
| 2-Butenal | 3,91 | 47,0 | 0,17 | 4,51E-05 | 4,99 | 44,4 | 5,72E-06 | 8,39E-06 | 0,41 [0,27 - 0,56] |
| Me1(MOme)2pyrrolidinec | 4,97 | 4630,2 | -0,49 | 5,71E-05 | 5,46 | 1000,0 | 0,08743 | 0,09528 | 0,38 [-0,07 - 0,84] |
| Me6oxo3pyridinecarboxylate | 5,13 | 1762,2 | 0,76 | 1,06E-04 | 3,17 | 1000,0 | 0,17535 | 0,17968 | 0,49 [-0,25 - 1,23] |
| 2-(methoxymethyl)-Furan | 4,75 | 28,6 | -0,29 | 1,11E-04 | 5,53 | 442,1 | 1,53E-05 | 6,18E-06 | 0,85 [0,58 - 1,13] |
| n-Pentanal | 4,24 | 48,1 | 0,27 | 1,12E-04 | 4,97 | 35,1 | 3,81E-05 | 7,69E-05 | 0,52 [0,31 - 0,74] |
| Tetra Me carboxylate | 5,73 | 346,3 | 1,28 | 1,22E-04 | 1,62 | 1000,0 | 1,00000 | 0,81699 | 0,15 [-1,57 - 1,88] |
| Gamma-HCH | 3,60 | 268,9 | 0,58 | 1,26E-04 | 1,72 | 1000,0 | 0,12500 | 0,17337 | 1,03 [-0,81 - 2,87] |
| Dibutyl phthalate | 3,14 | 6,6 | -1,11 | 1,42E-04 | 6,11 | 91,7 | 0,43043 | 0,38688 | -0,20 [-0,68 - 0,28] |
| Me 8-oxononaoate | 6,35 | 7215,3 | 0,40 | 2,03E-04 | 7,26 | 32,0 | 0,00122 | 0,00080 | 0,37 [0,19 - 0,55] |
| Tetradecanoic acid | 3,83 | 38,2 | 0,86 | 3,34E-04 | 0,48 | 1000,0 | 0,50000 | 0,00012 | 0,07 [0,07 - 0,07] |
| 2-MO-N-me hippurate | 4,30 | 1000,0 | 0,40 | 3,87E-04 | 2,41 | 1000,0 | 0,62500 | 0,47048 | 0,12 [-0,36 - 0,61] |
| Methyl-5-oxoproline | 6,31 | 2922,7 | 0,86 | 4,94E-04 | 3,72 | 1000,0 | 0,29688 | 0,20953 | 0,78 [-0,58 - 2,13] |
| Me-3OH-2-methylpentanoate | 4,41 | 67,3 | 0,33 | 6,61E-04 | 5,01 | 1000,0 | 0,00336 | 0,00629 | 0,50 [0,16 - 0,83] |
| TetraMe diMOiminobutandioate | 6,46 | 1000,0 | 0,64 | 7,11E-04 | 0,48 | 1000,0 | 0,25000 | 0,16869 | 1,40 [-1,45 - 4,25] |
| Dimethyl oxalacetate | 5,29 | 702,2 | 0,31 | 8,13E-04 | 6,50 | 11633,8 | 0,27444 | 0,15595 | 0,33 [-0,14 - 0,81] |
| FAME C19:0 | 2,60 | 27,8 | -0,58 | 9,37E-04 | 2,80 | 42,2 | 0,08977 | 0,05925 | -0,20 [-0,40 - 0,01] |
| 2,4(E,E)-Decadienal | 4,45 | 189,4 | 0,46 | 9,50E-04 | 6,06 | 4890,6 | 0,00143 | 0,00153 | 0,53 [0,23 - 0,83] |
| FAME trans-C12:1n9 | 4,08 | 346,3 | 0,45 | 0,0010 | 5,51 | 1000,0 | 0,02299 | 0,03094 | 0,59 [0,06 - 1,12] |
| 1,3-Dimethyl parabanoate | 3,80 | 222,0 | 0,87 | 0,0011 | 4,18 | 8081,1 | 0,03623 | 0,09711 | 0,25 [-0,05 - 0,56] |
| FAME i-C14:0-2,6,10Me | 3,81 | 27,3 | 0,51 | 0,0011 | 3,44 | 167,7 | 0,67770 | 0,81521 | 0,07 [-0,54 - 0,67] |
| Pentadecanoic acid | 3,39 | 76,4 | 0,27 | 0,0012 | 1,74 | 1000,0 | 1,00000 | 0,97022 | 0,00 [-0,20 - 0,20] |
| N-Methyl-2-propenamide | 4,31 | 275,7 | -0,41 | 0,0013 | 5,17 | 892,6 | 0,29425 | 0,30996 | 0,13 [-0,13 - 0,39] |
| trans-Methyl-2-butenoate | 4,50 | 38,0 | -0,21 | 0,0017 | 5,80 | 307,5 | 8,20E-05 | 7,68E-05 | 0,35 [0,21 - 0,50] |
| Propanamide | 4,38 | 266,4 | 0,26 | 0,0018 | 5,29 | 28,3 | 0,00073 | 0,00038 | 0,78 [0,43 - 1,13] |
| cis-Methyl 3-octenoate | 4,77 | 96,6 | 0,22 | 0,0025 | 6,22 | 23,5 | 2,67E-05 | 2,45E-05 | 0,51 [0,32 - 0,71] |
| FAME trans-C18:1n9 | 8,40 | 360757,7 | -0,20 | 0,0028 | 8,64 | 3078,8 | 0,00336 | 0,00355 | 0,23 [0,09 - 0,38] |
| Dimethyl proline | 3,30 | 65,1 | 0,37 | 0,0028 | 4,21 | 1000,0 | 0,00076 | 0,00068 | 0,45 [0,22 - 0,67] |
| trans-2-Pentenal | 3,86 | 11,8 | 0,13 | 0,0029 | 4,42 | 44,4 | 0,00052 | 0,00036 | 0,43 [0,22 - 0,63] |
| FAME C17:0-d | 6,37 | 27509,0 | 0,59 | 0,0034 | 3,58 | 77,2 | 0,12021 | 0,08119 | -0,84 [-1,80 - 0,12] |
| 3-Decanone | 3,02 | 7,4 | 0,21 | 0,0043 | 3,23 | 23,4 | 3,81E-05 | 6,33E-06 | 1,06 [0,71 - 1,42] |
| Dibenzo thiophene | 2,89 | 7,7 | -0,31 | 0,0044 | 0,48 | 1000,0 | 0,43750 | 0,31822 | 0,39 [-0,52 - 1,30] |
| Benzaldehyde | 4,65 | 20,9 | 0,18 | 0,0046 | 6,27 | 40,0 | 9,54E-06 | 9,36E-06 | 0,31 [0,20 - 0,41] |
| Trimethyl cis-aconitate | 4,07 | 1111,6 | -0,32 | 0,0058 | 5,83 | 38954,8 | 0,10416 | 0,39399 | 0,21 [-0,30 - 0,72] |
| 2,5-Piperidindione | 6,61 | 1370,1 | -0,19 | 0,0066 | 6,26 | 218,1 | 0,12309 | 0,07765 | 0,29 [-0,04 - 0,62] |
| FAME C15:0 | 7,02 | 2679,1 | 0,28 | 0,0068 | 7,28 | 2172,8 | 0,36828 | 0,60821 | -0,30 [-1,53 - 0,92] |
| 2-Pyrrolidinone | 5,65 | 1352,4 | 0,62 | 0,0070 | 6,37 | 443,1 | 0,00071 | 0,00093 | 0,50 [0,23 - 0,77] |
| 5-Me-2-Furanmethanol | 4,56 | 53,5 | 0,18 | 0,0072 | 6,51 | 381,2 | 9,54E-06 | 3,93E-05 | 0,55 [0,34 - 0,77] |
| Dimethylsulfoxide | 6,88 | 6,7 | 0,32 | 0,0075 | 8,19 | 20,1 | 0,03277 | 0,03892 | 0,11 [0,01 - 0,21] |
| 2,4,6-TriMO Pyrimidine | 5,01 | 335,0 | -0,32 | 0,0077 | 4,96 | 1000,0 | 0,01387 | 0,01226 | 0,53 [0,13 - 0,94] |
| alpha-Patchoulene | 4,94 | 1000,0 | 0,21 | 0,0085 | 5,60 | 1000,0 | 0,14746 | 0,29084 | 0,17 [-0,17 - 0,51] |
| Methyl (2Z)-4-MO-2-butenoate | 2,43 | 1000,0 | -0,29 | 0,0085 | 0,48 | 1000,0 | 1,00000 | 0,52272 | 0,01 [-0,09 - 0,10] |
| 2,3-Butandione | 3,50 | 8,5 | -0,41 | 0,0089 | 0,48 | 1000,0 | 0,25000 | 0,01069 | 0,43 [0,24 - 0,62] |
| Diacetamide | 4,16 | 51,5 | 0,24 | 0,0104 | 4,15 | 615,2 | 0,03906 | 0,03182 | 0,25 [0,03 - 0,48] |
| Safrole | 3,35 | 346,3 | 0,30 | 0,0108 | 1,85 | 1000,0 | 0,68750 | 0,55726 | 0,15 [-0,44 - 0,74] |
| Me cyclopentanone2carboxylate | 0,00 | 389,7 | -0,42 | 0,0145 | 0,48 | 1000,0 | 0,25000 | 0,18347 | 0,99 [-1,14 - 3,13] |
| Trimethylamino OH butyrate | 6,29 | 449,7 | -0,25 | 0,0148 | 6,76 | 92,8 | 8,20E-05 | 0,00026 | 0,61 [0,33 - 0,90] |
| 2-Acetylfuran | 4,22 | 7,8 | 0,11 | 0,0168 | 5,46 | 99,4 | 0,00121 | 0,00071 | 0,83 [0,40 - 1,26] |
| cis-Me-4-oxo-2-pentenoate | 4,10 | 6,4 | -0,18 | 0,0187 | 5,80 | 32,8 | 1,34E-05 | 2,35E-06 | 0,34 [0,23 - 0,44] |
| Tri Me Homocystein | 6,27 | 1000,0 | 0,29 | 0,0190 | 0,48 | 1000,0 | 0,87500 | 0,68932 | -0,08 [-0,68 - 0,52] |
| 3,4-Di-MO-cinnamaldehyde | 0,00 | 142,1 | -0,90 | 0,0218 | 2,87 | 96,6 | 0,00015 | 0,00044 | 0,53 [0,28 - 0,79] |
| n-Hexanal | 5,13 | 39,1 | 0,18 | 0,0224 | 6,15 | 38,6 | 3,62E-05 | 8,06E-06 | 0,50 [0,33 - 0,67] |
| 2-Oxobutanoic acid | 0,00 | 28,8 | 0,25 | 0,0256 | 3,87 | 39,1 | 0,02667 | 0,02274 | 0,27 [0,04 - 0,50] |
| 2-Hydroxy acetophenone | 4,51 | 1146,4 | 0,21 | 0,0266 | 6,44 | 2937,4 | 0,00039 | 0,00028 | 0,35 [0,19 - 0,52] |
| Butanedial | 2,77 | 11,3 | -0,28 | 0,0291 | 3,62 | 24,5 | 0,00097 | 0,00138 | 0,32 [0,14 - 0,50] |
| 2-Nitrotoluene | 4,94 | 1000,0 | 0,28 | 0,0302 | 5,51 | 1000,0 | 0,02667 | 0,02625 | 0,39 [0,05 - 0,73] |
| Formamide | 4,95 | 1870,2 | 0,39 | 0,0304 | 5,99 | 170,8 | 0,00169 | 0,00287 | 0,48 [0,19 - 0,78] |
| Trimethyl trans-aconitate | 4,73 | 1964,2 | -0,18 | 0,0306 | 6,09 | 30022,8 | 0,00658 | 0,00459 | 0,55 [0,19 - 0,91] |
| 4-Ethylphenol | 3,41 | 6,2 | 0,42 | 0,0315 | 3,33 | 35,0 | 0,57984 | 0,33568 | 0,27 [-0,31 - 0,85] |
| 2-Methoxy-3-isobutyl pyrazine | 0,00 | 346,3 | NA | 0,0324 | 3,68 | 1000,0 | 0,15625 | 0,10361 | 0,86 [-0,24 - 1,95] |
| N-Methyl formamide | 5,47 | 8486,1 | 0,36 | 0,0358 | 6,52 | 705,7 | 0,00422 | 0,00390 | 0,39 [0,14 - 0,64] |
| trans-2-Octenal | 4,47 | 66,5 | 0,10 | 0,0438 | 5,68 | 58,9 | 0,00085 | 0,00065 | 0,64 [0,31 - 0,97] |
| 4-Methylphenol | 0,00 | 156,8 | -0,59 | 0,0473 | 4,46 | 68,7 | 0,58301 | 0,56331 | 0,14 [-0,38 - 0,67] |
| Chinoline | 4,94 | 1936,4 | 0,38 | 0,0501 | 5,56 | 18024,6 | 0,00015 | 0,00011 | 0,42 [0,25 - 0,59] |
| Methyl-4-oxo-2-pentenoate | 4,27 | 16,6 | -0,12 | 0,0538 | 4,68 | 32,7 | 0,00336 | 0,00587 | 0,31 [0,10 - 0,51] |
| beta-Ionone | 5,91 | 6473,0 | 0,22 | 0,0541 | 5,69 | 30920,0 | 0,24621 | 0,23616 | 0,34 [-0,24 - 0,91] |
| FAME i-C16:0 | 3,29 | 8,2 | -0,47 | 0,0555 | 5,18 | 1646,7 | 0,03036 | 0,05933 | -0,59 [-1,21 - 0,03] |
| Dimethyl aspartate | 4,67 | 23,0 | -0,17 | 0,0587 | 5,73 | 204,1 | 8,20E-05 | 0,00011 | 0,55 [0,31 - 0,79] |
| Methyl-3-OH-hexanoate | 4,20 | 3455,6 | 0,77 | 0,0588 | 0,48 | 59,3 | 0,62500 | 0,51426 | 0,45 [-1,31 - 2,22] |
| Trimethyl isoserine | 7,27 | 1982,9 | 0,21 | 0,0621 | 5,37 | 1000,0 | 0,66855 | 0,40765 | 0,26 [-0,39 - 0,90] |
| N-Formylmorpholine | 4,99 | 150,0 | -0,23 | 0,0621 | 0,48 | 136,2 | 0,84375 | 0,76990 | 0,33 [-2,41 - 3,06] |
| FAME i-C10:0-4,6Me | 0,00 | 5,8 | -0,60 | 0,0631 | 2,50 | 58,3 | 0,92188 | 0,79471 | 0,08 [-0,56 - 0,71] |
| Dibenzofuran | 4,67 | 6527,1 | -0,53 | 0,0644 | 6,14 | 117,9 | 0,15137 | 0,19064 | 0,13 [-0,08 - 0,35] |
| Dimethyl terephthalate | 4,20 | 1049,1 | -1,17 | 0,0644 | 6,14 | 161,0 | 0,76953 | 0,70785 | -0,08 [-0,56 - 0,39] |
| Tetramethyl 2-Me aspartate | 5,53 | 1829,1 | 0,18 | 0,0645 | 6,41 | 3222,7 | 0,00516 | 0,00408 | 0,88 [0,33 - 1,43] |
| Acetamide | 5,67 | 17185,7 | 0,30 | 0,0670 | 6,82 | 3162,6 | 0,00199 | 0,00200 | 0,41 [0,17 - 0,65] |
| Ethylene brassylate | 1,77 | 9,9 | -0,26 | 0,0694 | 2,08 | 1000,0 | 0,16735 | 0,29339 | -0,08 [-0,23 - 0,08] |
| 1,4-Dimethoxy benzene | 5,29 | 1520,9 | -0,12 | 0,0695 | 6,51 | 15764,7 | 0,27736 | 0,32273 | 0,11 [-0,12 - 0,34] |
| 2,2-DiMe-propanamide | 3,44 | 113,2 | 0,18 | 0,0697 | 3,73 | 97,2 | 0,80396 | 0,80153 | -0,03 [-0,29 - 0,23] |
| Trimethyl iminodiacetate | 5,53 | 3026,8 | -0,16 | 0,0710 | 4,51 | 1000,0 | 0,43750 | 0,35479 | 0,50 [-0,75 - 1,75] |
| Valerolactone | 5,80 | 1072,9 | 0,18 | 0,0728 | 5,53 | 54,2 | 2,67E-05 | 7,66E-05 | 0,67 [0,39 - 0,95] |
| FAME trans-C14:1n11 | 5,31 | 3466,4 | 0,41 | 0,0736 | 5,95 | 9853,4 | 0,97995 | 0,76828 | 0,10 [-0,63 - 0,84] |
| Butanedinitrile | 1,64 | 34,9 | 0,02 | 0,0750 | 0,48 | 23,2 | 0,50000 | 0,23717 | -0,55 [-3,28 - 2,18] |
| 4-Cyanopyridine | 3,50 | 46,0 | 0,10 | 0,0767 | 4,50 | 158,6 | 0,00121 | 0,00190 | 0,32 [0,13 - 0,50] |
| Methyl 4-methoxybenzoate | 6,42 | 729,5 | -0,15 | 0,0780 | 7,31 | 14518,8 | 0,00039 | 0,00058 | 0,41 [0,20 - 0,61] |
| 2(5H)-Furanone | 5,25 | 127,8 | 0,16 | 0,0834 | 0,48 | 49,7 | 0,50000 | 0,22973 | 0,39 [-0,59 - 1,37] |
| alpha-Terpineol | 4,47 | 17,3 | 0,28 | 0,0890 | 0,48 | 18,1 | 0,87500 | 0,93552 | -0,03 [-1,17 - 1,11] |
| Methyl salicylate | 4,47 | 199,0 | 0,14 | 0,1098 | 4,93 | 2688,5 | 0,00169 | 0,00097 | 0,71 [0,33 - 1,09] |
| Dimethylsulfone | 5,43 | 78,3 | -0,13 | 0,1159 | 7,64 | 27,9 | 6,29E-05 | 0,00011 | 0,27 [0,15 - 0,39] |
| Tetramethyl aminoadipate | 6,00 | 17332,5 | 0,13 | 0,1170 | 5,94 | 412,4 | 0,04248 | 0,12817 | 0,79 [-0,27 - 1,84] |
| Methyl-9-oxononaoate | 5,74 | 573,7 | 0,80 | 0,1182 | 4,33 | 125,8 | 0,00134 | 0,00128 | 1,00 [0,46 - 1,55] |
| 2-Methyl-5-oxoprolinemethylester | 6,97 | 2355,6 | -0,12 | 0,1201 | 6,17 | 305704,7 | 0,19009 | 0,13234 | 0,42 [-0,14 - 0,97] |
| FAME i-C14:0-12Me | 3,75 | 34,9 | -0,24 | 0,1213 | 4,89 | 45,6 | 0,20245 | 0,12167 | -0,29 [-0,66 - 0,08] |
| Dime 2,3-dime butandioate | 0,00 | 12,6 | -0,66 | 0,1230 | 0,48 | 23,6 | 0,06250 | 0,00835 | 0,96 [0,41 - 1,50] |
| cis-Methyl 3-nonenoate | 3,68 | 8,1 | 0,24 | 0,1331 | 5,35 | 1000,0 | 0,00342 | 0,00593 | 0,36 [0,13 - 0,60] |
| 2,3-Octanedione | 3,82 | 38,7 | NA | 0,1345 | 4,76 | 1000,0 | 0,06250 | 0,01467 | 0,50 [0,16 - 0,84] |
| O,N-Dimethylcarbamate(CO2/NH3) | 5,26 | 259,7 | -0,10 | 0,1385 | 7,06 | 5072,2 | 0,00121 | 0,00079 | 0,39 [0,18 - 0,59] |
| beta-Ionone epoxide | 3,32 | 402,8 | 0,27 | 0,1441 | 3,61 | 1000,0 | 0,29688 | 0,44698 | 0,33 [-0,66 - 1,32] |
| 2-Ethylidenemalononitrile | 6,08 | 65065,7 | 0,25 | 0,1452 | 6,25 | 91468,7 | 0,95298 | 0,60006 | -0,04 [-0,20 - 0,12] |
| 2,4-Dimethoxy pyrimidine | 1,15 | 13,5 | NA | 0,1569 | 0,48 | 1000,0 | 0,50000 | 0,45027 | 0,56 [-5,56 - 6,69] |
| Acetic acid | 0,00 | 6,7 | 0,31 | 0,1575 | 1,56 | 26,8 | 0,12500 | 0,12254 | 1,16 [-0,49 - 2,81] |
| 3,5-Dimethoxytoluene | 5,14 | 999,9 | -0,15 | 0,1649 | 4,16 | 1000,0 | 0,37546 | 0,30710 | 0,14 [-0,14 - 0,42] |
| FAME C14:0, 2-OH | 4,03 | 536,1 | 0,12 | 0,1771 | 0,48 | 1000,0 | 0,87500 | 0,96906 | 0,02 [-1,74 - 1,78] |
| Hydroxy acetic acid | 3,52 | 302,0 | -0,25 | 0,1841 | 5,19 | 611,7 | 0,85522 | 0,59724 | 0,06 [-0,19 - 0,31] |
| Methyl-3-methoxypropionate | 2,99 | 5,4 | 0,07 | 0,1900 | 0,48 | 40,5 | 0,25000 | 0,00127 | 0,44 [0,37 - 0,50] |
| FAME C12:0^*^ | 6,89 | 1629,2 | 0,32 | 0,1917 | 8,44 | 54,4 | 0,06958 | 0,12033 | 0,50 [-0,14 - 1,14] |
| 1-Methyl-2-oxazolidinone | 5,71 | 12,3 | 0,08 | 0,2019 | 7,20 | 387,4 | 0,00102 | 0,00080 | 0,27 [0,13 - 0,42] |
| Me 4-oxo-2-heptenedioate | 4,72 | 1247,1 | -0,18 | 0,2094 | 4,92 | 84,4 | 0,00977 | 0,02485 | 1,00 [0,16 - 1,85] |
| Dimethyl butanedioate | 6,96 | 371,0 | 0,09 | 0,2128 | 7,58 | 314,7 | 4,77E-05 | 0,00057 | 0,51 [0,25 - 0,77] |
| Furfurylmethanolacetate | 3,07 | 24,6 | -0,12 | 0,2162 | 4,70 | 32,1 | 0,70118 | 0,57475 | -0,08 [-0,36 - 0,21] |
| Diphenyl ether | 0,00 | 24,5 | NA | 0,2232 | 6,44 | 27451,2 | 6,29E-05 | 4,73E-05 | 0,51 [0,30 - 0,71] |
| Trimethyl-O-ethylhomoserine | 6,89 | 1263,3 | 0,09 | 0,2344 | 7,01 | 1000,0 | 0,12500 | 0,06202 | 0,71 [-0,07 - 1,49] |
| Butanamide | 1,08 | 120,8 | 1,21 | 0,2379 | 4,78 | 1000,0 | 0,07813 | 0,05866 | 0,88 [-0,04 - 1,80] |
| FAME C14:0^*^ | 0,00 | 271,7 | -0,40 | 0,2389 | 8,85 | 172,8 | 0,36828 | 0,10083 | -0,69 [-1,53 - 0,15] |
| Pristan (i-C19) | 4,62 | 10,4 | -0,10 | 0,2448 | 0,48 | 24,8 | 0,50000 | 0,50950 | 0,32 [-1,43 - 2,08] |
| Dimethyl glutarate | 6,81 | 763,6 | 0,39 | 0,2467 | 7,44 | 6105,5 | 1,91E-06 | 5,58E-05 | 0,90 [0,54 - 1,27] |
| 1-Cyclohexenyl acetic acid | 3,45 | 19,7 | -0,13 | 0,2631 | 3,17 | 1000,0 | 0,62500 | 0,44116 | 0,23 [-0,52 - 0,98] |
| 1-Pentyl pyrrole | 4,31 | 553,7 | 0,20 | 0,2689 | 3,18 | 40,0 | 0,73438 | 0,80643 | 0,09 [-0,72 - 0,89] |
| Trimethyl phosphate | 4,55 | 1000,0 | -0,07 | 0,2706 | 5,18 | 1000,0 | 0,00610 | 0,00396 | 0,36 [0,14 - 0,59] |
| 1,3-Dime-5-MOMe uracil | 1,88 | 31,8 | -0,33 | 0,2747 | 4,84 | 1000,0 | 0,25000 | 0,22979 | 0,89 [-1,36 - 3,14] |
| O-Methylcarbamate (CO2/NH3) | 5,22 | 722,3 | 0,06 | 0,2781 | 6,91 | 2121,6 | 0,01069 | 0,00817 | 0,32 [0,09 - 0,55] |
| TriMe propan123tricarboxylate | 3,52 | 8,1 | -0,12 | 0,2791 | 0,48 | 1000,0 | 0,62500 | 0,56374 | 0,21 [-0,82 - 1,24] |
| Tetramethyl aminomalonate | 4,60 | 1000,0 | -0,07 | 0,2807 | 5,66 | 1000,0 | 0,00516 | 0,00337 | 0,40 [0,15 - 0,64] |
| trans-Methylcinnamate | 4,58 | 63,5 | 0,10 | 0,2884 | 5,85 | 3373,6 | 0,00385 | 0,00956 | 0,47 [0,13 - 0,81] |
| Phenylacetaldehyde | 0,00 | 33,2 | -0,46 | 0,2907 | 4,83 | 249,7 | 0,01531 | 0,00974 | 0,21 [0,06 - 0,37] |
| 2,4(E,E)-Octadienal | 4,46 | 9,8 | 0,20 | 0,2939 | 5,34 | 1000,0 | 0,00258 | 0,00203 | 0,48 [0,20 - 0,75] |
| 5-Ethyl-2(5H)-furanone | 3,35 | 20,4 | 0,32 | 0,2966 | 0,48 | 172,5 | 1,00000 | 0,70182 | -0,09 [-2,41 - 2,22] |
| Tetramethyl-2-me succinate | 4,15 | 97,6 | 0,13 | 0,2971 | 5,32 | 1000,0 | 0,01099 | 0,02373 | 0,36 [0,06 - 0,67] |
| FAME trans-C18:2n7,10 | 0,00 | 8,1 | 0,20 | 0,2985 | 0,48 | 611,5 | 0,25000 | 0,12552 | 0,43 [-0,29 - 1,15] |
| Methyl 2,4 diMO orotate | 3,40 | 1000,0 | -0,19 | 0,3204 | 2,47 | 1000,0 | 0,25000 | 0,04843 | 0,40 [0,01 - 0,80] |
| Naphthalene | 3,20 | 5,8 | 0,13 | 0,3225 | 4,43 | 59,5 | 1,53E-05 | 2,45E-05 | 0,76 [0,48 - 1,03] |
| 2,6(E,E)-Heptadienal | 3,54 | 102,0 | -0,05 | 0,3297 | 4,84 | 95,8 | 6,29E-05 | 6,54E-05 | 0,79 [0,47 - 1,12] |
| Methyl-8-oxo octanoate | 3,96 | 315,8 | -0,38 | 0,3309 | 4,47 | 55,8 | 0,42578 | 0,23231 | 0,31 [-0,24 - 0,86] |
| 2H-Pyran-2-one | 4,01 | 18,0 | 0,02 | 0,3317 | 4,78 | 20,1 | 0,08969 | 0,03961 | 0,11 [0,01 - 0,22] |
| Hydroxyacetone | 3,89 | 30,3 | 0,10 | 0,3386 | 4,67 | 1000,0 | 0,14746 | 0,17704 | 0,63 [-0,34 - 1,60] |
| 2,4(E,E)-Nonadienal | 0,00 | 38,6 | -0,14 | 0,3626 | 5,87 | 880,0 | 0,29425 | 0,16951 | 0,16 [-0,08 - 0,41] |
| Methyl 3,4-dimethoxybenzoate | 0,00 | 5,4 | NA | 0,3638 | 4,81 | 38,1 | 0,10416 | 0,07355 | 0,42 [-0,04 - 0,89] |
| N-Methyl acetamide | 5,62 | 59646,4 | -0,07 | 0,3884 | 5,99 | 639,3 | 0,00121 | 0,00058 | 0,38 [0,19 - 0,57] |
| Methylformiate C1 | 0,00 | 346,3 | -1,27 | 0,4001 | 0,48 | 21,0 | 0,50000 | 0,08598 | -0,09 [-0,24 - 0,06] |
| Methyl cyanoacetate | 0,00 | 346,3 | NA | 0,4222 | 1,65 | 1000,0 | 0,50000 | 0,25934 | -0,17 [-0,63 - 0,29] |
| FAME a-C15-12Me | 3,22 | 59,4 | -0,12 | 0,4229 | 5,00 | 188,1 | 0,49801 | 0,69735 | -0,13 [-0,82 - 0,56] |
| Methyl methylenamine | 5,08 | 25,4 | -0,30 | 0,4264 | 5,83 | 33,3 | 0,14291 | 0,09901 | 0,33 [-0,07 - 0,73] |
| Trimethyl glycine | 0,00 | 346,3 | 0,13 | 0,4322 | 3,88 | 1000,0 | 0,04688 | 0,03051 | 0,29 [0,04 - 0,53] |
| Trimethyl homoserine | 6,16 | 678,4 | -0,08 | 0,4364 | 6,01 | 1000,0 | 0,94531 | 0,28782 | 0,75 [-0,79 - 2,28] |
| Dimethyl iminodiacetate | 0,00 | 326,6 | NA | 0,4484 | 0,48 | 1000,0 | 1,00000 | 0,56784 | 1,04 [-15,40 - 17,49] |
| 2-Methylchinolin | 3,88 | 2989,5 | 0,51 | 0,4597 | 3,66 | 1000,0 | 0,82031 | 0,86925 | -0,05 [-0,77 - 0,66] |
| Methyl 2-phenylacetate | 4,56 | 80,7 | -0,04 | 0,4599 | 5,66 | 382,9 | 0,00169 | 0,00430 | 0,40 [0,14 - 0,65] |
| 1-Cyclohexenecarboxylicacid | 3,66 | 17,6 | 0,63 | 0,4604 | 5,64 | 329,9 | 0,00315 | 0,00282 | 0,56 [0,22 - 0,91] |
| FAME i-C15-13Me | 3,08 | 9,3 | -0,26 | 0,4694 | 6,16 | 4255,1 | 0,00365 | 0,01295 | -0,73 [-1,28 - -0,17] |
| 3-Me-2-oxo-pyrane-6-acid | 3,21 | 78,2 | 0,26 | 0,4859 | 5,35 | 36,1 | 0,06396 | 0,05538 | 0,29 [-0,01 - 0,59] |
| Methyl 3-oxopentanoate | 0,00 | 346,3 | -0,03 | 0,5133 | 0,48 | 1000,0 | 0,12500 | 0,03211 | 0,53 [0,09 - 0,98] |
| FAME a-C13:0-10Me | 3,20 | 13,3 | 0,09 | 0,5201 | 3,89 | 54,2 | 0,59489 | 0,43130 | 0,19 [-0,30 - 0,68] |
| Dimethylmalonate | 4,40 | 13,6 | 0,04 | 0,5387 | 4,95 | 386,3 | 0,04199 | 0,04592 | 0,17 [0,00 - 0,34] |
| N,N-Dimethylurea | 6,57 | 373247,0 | 0,06 | 0,5441 | 6,85 | 1524,0 | 0,10838 | 0,24943 | 0,27 [-0,21 - 0,74] |
| Methyl alanine | 3,24 | 346,3 | 0,04 | 0,5518 | 1,96 | 1000,0 | 0,25000 | 0,17968 | 0,75 [-0,84 - 2,35] |
| 2-Methoxyphenol | 3,26 | 346,3 | -0,09 | 0,5548 | 4,71 | 42,4 | 0,28416 | 0,40171 | 0,11 [-0,16 - 0,38] |
| FAME C13:0 | 4,19 | 55,7 | -0,11 | 0,5550 | 5,89 | 1094,9 | 0,29425 | 0,31137 | 0,21 [-0,21 - 0,64] |
| Methyl nitrite (NO) | 1,73 | 346,3 | -0,20 | 0,5675 | 3,64 | 18,1 | 7,63E-05 | 9,00E-05 | 0,53 [0,31 - 0,75] |
| N-Methyl diuron | 3,03 | 8,4 | -0,09 | 0,5704 | 3,49 | 1000,0 | 0,43750 | 0,55532 | 0,12 [-0,38 - 0,62] |
| Dimethyl adipate | 5,93 | 118,7 | 0,12 | 0,5779 | 7,03 | 815,0 | 3,81E-06 | 7,69E-05 | 0,59 [0,35 - 0,84] |
| 3-Phenoxytoluene | 3,92 | 29,2 | -0,02 | 0,5813 | 4,07 | 1000,0 | 0,69531 | 0,98391 | -0,01 [-0,70 - 0,69] |
| Methyl undecanoate C11:0 | 3,90 | 14,5 | 0,06 | 0,5836 | 5,18 | 51,9 | 8,20E-05 | 0,00027 | 0,99 [0,52 - 1,45] |
| 1-Methyl indole | 0,00 | 21,5 | NA | 0,6060 | 3,32 | 89,2 | 0,00557 | 0,00586 | 0,22 [0,07 - 0,37] |
| Methyl 2-methyllactate | 3,14 | 32,3 | 0,07 | 0,6187 | 4,70 | 1000,0 | 0,06250 | 0,09536 | 0,19 [-0,05 - 0,44] |
| FAME a-C17:0 | 5,46 | 2394,3 | -0,20 | 0,6350 | 5,97 | 10358,5 | 0,95633 | 0,51553 | 0,13 [-0,28 - 0,54] |
| Tri Me Hydantoin | 5,26 | 460,8 | -0,04 | 0,6367 | 6,14 | 511,2 | 8,20E-05 | 9,62E-05 | 0,37 [0,21 - 0,53] |
| Methyl-2-hydroxy acetate | 5,38 | 42,5 | -0,16 | 0,6488 | 5,62 | 3210,7 | 0,00486 | 0,00366 | 0,48 [0,18 - 0,79] |
| tr-Dimethyl-2-hexenedioate | 5,15 | 811,9 | 0,15 | 0,6738 | 6,25 | 104,1 | 3,62E-05 | 2,65E-05 | 1,29 [0,80 - 1,77] |
| 3-Me-2,5-oxazolidine-dione | 4,01 | 45,4 | 0,05 | 0,6792 | 0,48 | 2314,7 | 1,00000 | 0,52530 | 0,66 [-8,43 - 9,76] |
| 4-Methyl pentanoic acid | 3,90 | 8,0 | 0,37 | 0,7223 | 5,05 | 44,6 | 0,81250 | 0,52371 | 0,29 [-0,77 - 1,35] |
| 2,4-Dicyano-1-butene | 5,31 | 1563,7 | 0,41 | 0,7377 | 2,95 | 25,8 | 0,46094 | 0,19615 | 0,56 [-0,37 - 1,50] |
| Methyl lactate | 5,02 | 9,7 | 0,03 | 0,7515 | 5,81 | 40,5 | 0,02893 | 0,02026 | 0,26 [0,04 - 0,47] |
| trans-2-Heptenal | 3,85 | 28,0 | 0,02 | 0,7775 | 5,20 | 132,5 | 0,00032 | 0,00035 | 0,42 [0,22 - 0,63] |
| Di-Me-5-oxoproline | 0,00 | 346,3 | NA | 0,7820 | 0,48 | 1000,0 | 1,00000 | 0,51034 | 1,54 [-18,62 - 21,69] |
| 2,4-Dichloro-6-methylphenol | 0,00 | 6,2 | -0,10 | 0,8048 | 0,48 | 1000,0 | 0,25000 | 0,05635 | 1,73 [-0,12 - 3,58] |
| N-Meoxycarb-Proline-Me | 6,22 | 4209,6 | 0,02 | 0,8063 | 6,57 | 14856,9 | 0,00013 | 8,78E-05 | 0,64 [0,37 - 0,91] |
| Dimethylfumarate | 5,63 | 24,0 | 0,02 | 0,8183 | 6,31 | 1502,8 | 0,00271 | 0,00319 | 0,38 [0,14 - 0,61] |
| FAME cis-C12:1n9 | 4,99 | 1131,3 | -0,06 | 0,8255 | 5,60 | 541,1 | 0,76801 | 0,16240 | -0,44 [-1,08 - 0,20] |
| Isopropyl tetradecanoate | 3,84 | 22,8 | -0,04 | 0,8265 | 4,96 | 681,1 | 0,18187 | 0,68598 | 0,09 [-0,39 - 0,58] |
| 1,3-Dimethoxy benzene | 4,62 | 270,5 | 0,03 | 0,8266 | 6,70 | 12872,3 | 1,91E-06 | 4,93E-07 | 0,50 [0,36 - 0,64] |
| 1-Me-5-MO pyrimidin-2-one | 4,87 | 303,2 | -0,02 | 0,8420 | 6,86 | 548,8 | 0,43043 | 0,43582 | 0,05 [-0,08 - 0,18] |
| Di-Me-2-oxazolidone | 6,51 | 2042,6 | 0,00 | 0,8450 | 6,64 | 15470,6 | 0,07585 | 0,03966 | 0,37 [0,02 - 0,71] |
| Anisaldehyde | 5,78 | 326,9 | 0,02 | 0,8695 | 7,60 | 983,8 | 0,01531 | 0,01656 | 0,19 [0,04 - 0,35] |
| Me-methoxy formamide | 2,01 | 9,9 | 0,04 | 0,8882 | 2,72 | 1000,0 | 0,25000 | 0,16207 | 0,96 [-0,70 - 2,62] |
| 3-Octenal | 3,65 | 47,2 | -0,01 | 0,8956 | 3,49 | 33,6 | 1,91E-05 | 4,98E-06 | 0,39 [0,26 - 0,52] |
| Methyl acetoxyacetate | 4,92 | 143,1 | -0,04 | 0,9045 | 5,56 | 1000,0 | 0,10938 | 0,13542 | 0,23 [-0,10 - 0,57] |
| FAME cis-C20:3n6 | 2,74 | 26,8 | -0,06 | 0,9354 | 2,99 | 1000,0 | 0,54767 | 0,53707 | 0,05 [-0,11 - 0,21] |
| Methyl-3-OH-butanoate | 5,31 | 6,3 | 0,05 | 0,9397 | 6,60 | 1000,0 | 0,20313 | 0,13904 | 0,20 [-0,08 - 0,48] |
| Pyridine | 1,80 | 5,5 | -0,27 | 0,9469 | 3,01 | 1000,0 | 0,01069 | 0,01217 | 0,17 [0,04 - 0,29] |
| 3-Methyl-2-cyclopenten-1-one | 4,22 | 61,2 | 0,00 | 0,9522 | 5,72 | 1253,8 | 0,00013 | 7,79E-05 | 0,49 [0,29 - 0,70] |
| 2479Tetramethyl5-decyn47diol | 3,32 | 43,3 | -0,42 | 0,9618 | 4,94 | 1000,0 | 0,57031 | 0,48101 | 0,18 [-0,39 - 0,76] |
| FAME cis-C20:5n3 | 1,09 | 49,0 | 0,26 | 0,9795 | 3,40 | 1000,0 | 0,57060 | 0,42638 | 0,05 [-0,08 - 0,18] |
| 2-Hydroxybiphenyl | 2,93 | 36,8 | 0,03 | 0,9861 | 2,10 | 19,9 | 0,33960 | 0,52412 | -0,19 [-0,81 - 0,43] |
| Trimethyl cyanurate | 0,00 | 7,2 | -0,08 | 0,9968 | 5,29 | 2156,3 | 0,00831 | 0,01044 | 0,65 [0,17 - 1,13] |

## Supplementary Table 2 (Table S2)

Quantitation data from multiple extractions of a sample vial spiked with 75 ng azelaic acid dimethylester: The response factor was calculated as the sum of peak areas divided by the spiked amount of azelaic acid methyl ester (1.69×10^9^ counts / 75 ng = 2.25×10^7^ counts/ng). The “amount in fiber” is calculated as the peak area [counts] divided by 2.25×10^7^ counts/ng. The “amount in vial” is calculated as the amount spiked minus the amount in fiber.

|  | amount extracted | amount in fiber | amount in vial | ratio of amounts  extracted/in vial |
| --- | --- | --- | --- | --- |
| Extraction step | counts | ng | ng | counts/ng |
| 1 | 5.80E+08 | 26 | 75 | 7.73E+06 |
| 2 | 4.08E+08 | 18 | 49 | 8.28E+06 |
| 3 | 2.64E+08 | 12 | 31 | 8.50E+06 |
| 4 | 1.68E+08 | 7 | 19 | 8.71E+06 |
| 5 | 1.13E+08 | 5 | 12 | 9.56E+06 |
| 6 | 5.77E+07 | 3 | 7 | 8.49E+06 |
| 7 | 3.46E+07 | 2 | 4 | 8.18E+06 |
| 8 | 2.11E+07 | 1 | 3 | 7.85E+06 |
| 9 | 1.43E+07 | 1 | 2 | 8.15E+06 |
| 10 | 9.97E+06 | 0.4 | 1 | 8.93E+06 |
| 11 | 6.70E+06 | 0.3 | 0.7 | 9.95E+06 |
| 12 | 4.76E+06 | 0.2 | 0.4 | 1.27E+07 |
| 13 | 3.67E+06 | 0.2 | 0.2 | 2.25E+07 |
| Sum | 1.69E+09 |  | Mean | 9.96E+06 |

## Supplementary Table 3 (Table S3)

Rat (r) and human (h) primers and probes used for TaqMan PCR: Abbreviations: PCα1(I) – procollagen alpha 1(I); αSMA – alpha smooth muscle actin; TIMP-1 – tissue inhibitor of matrix metalloproteinase-1; TGF β – transforming growth factor beta; MMP – matrix metalloproteinase; GAPDH - glycerinaldehyd-3-phosphat-dehydrogenase; *MMP-13 (r), - ready to use Taqman assays from Applied Biosystems (Rotkreuz, Switzerland) for which the sequences were not provided.

| Target gene | 5´-Primer | Probe | 3´-Primer |
| --- | --- | --- | --- |
| PCα1(I) (r) | TCCGGCTCCTGCTCCTCTTA | TTCTTGGCCATGCGTCAGGAGGG | GTATGCAGCTGACTTCAGGGATGT |
| αSMA (r) | GCTGACAGGATGCAGAAGGA | CACCATGAAGATCAAGATTATTGCTCCTCCAG | GCCGATCCAGACAGAATATTTG |
| TIMP-1 (r) | TCCTCTTGTTGCTATCATTGATAGCTT | TTCTGCAACTCGGACCTGGTTATAAGG | CGCTGGTATAAGGTGGTCTCGAT |
| GAPDH (r) | CTGCCAAGTATGATGACATCAAGAA | TCGGCCGCCTGCTTCACCA | AGCCCAGGATGCCCTTTAGT |
| PCα1(I) (h) | CAGCCGCTTCACCTACAG | TGGCTGCACGAGTCACACCGG | GGTTTTGTATTCAATCACTGTCTT |
| GAPDH (h) | CCAGGAAATGAGCTTGACAAAGTT | TCGTTGAGGGCAATGCCAGCC | CTCCTCCACCTTTGACGCTG |

# Supplementary Figures

## Supplementary Figure 1 (Figure S1)

Example of analyte identification using GC/MS with chemical ionisation (CI) using water as reactent gas: The figure shows the CI mass spectrum of azelainic acid dimethylester (MW 216 ionized by addition of a proton = m/z 217 = MH^+^, and a fragment m/z 185 = MH^+^-CH_3_OH). The x-axis shows the mass (charge z = 1). The y-axis shows the relative amounts of the formed ions by ionization and fragmentation.


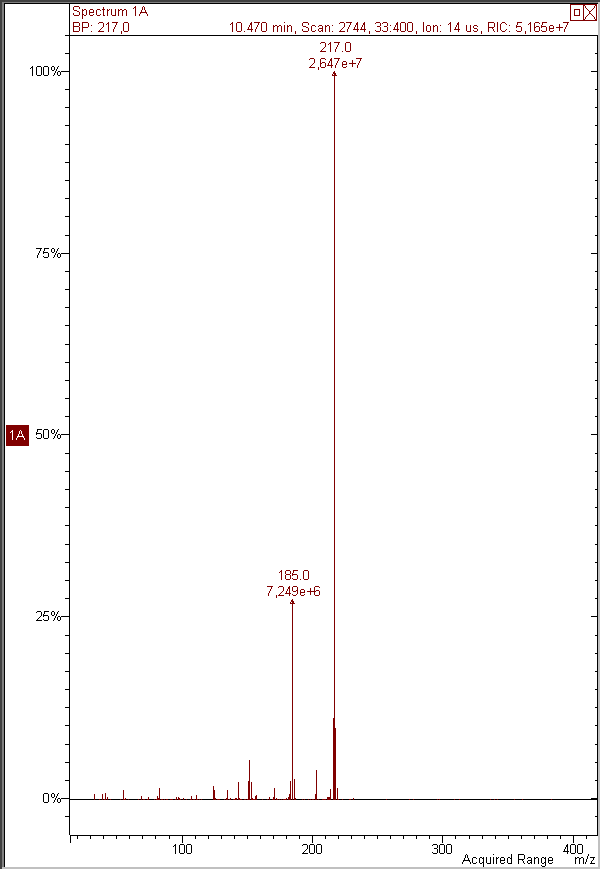


## Supplementary Figure 2 (Figure S2)

The Figure A shows the peak areas for 13 extraction steps of a vial, spiked with 75 ng of Azelainic acid dimethylester. In Figure B the linear correlation is shown between amount of analyte in the sample vial and the extracted peak area

## Supplementary Figure 3 (Figure S3)

DNA-synthesis of CFSC-2G (panel A) and MMNK-1 cells (panel B) cells as measured by BrDU incorporation using different concentrations of HAzPC. The bars show the extinction readings with the respective standard deviations. P-values for the comparison (two-sided t-test) to the control cells are provided for significant groups.

## Supplementary Figure 4 (Figure S4)

Analysis of mRNA expression using real-time PCR in CFSC-2G cells exposed to different concentrations of HAzPC. P-values for the comparison (two-sided t-test) to the control cells are provided for significant groups meeting the p<0.05 threshold.

# Supplementary Methods

## Preparation of phospholipid and triglyceride fractions

For separation of phospholipids from triglycerides, a modified technique described by Vandana et al. (S1) was applied. Ten milligrams of frozen ground tissue were supplemented with 100 µl of ice-cold acetone, transferred into a GC vial and incubated at 4°C for one hour at gentle agitation. After short centrifugation at 4°C the supernatant, rich in non-polar lipids (triglycerides, cholesterol) (S2) was transferred into a new GC vial. The incubation was repeated with another 100 µl volume of acetone, the supernatant was pooled with the first one and supplemented with 40 µl TMSH (25 %) following incubation for 15 min at 100°C as described above. The GC vial containing the tissue fragments with acetone insoluble phospholipids was supplemented with 40 µl TMSH (25 %) following incubation for 15 min at 100°C in the way described before. Theses samples were stored at room temperature until assayed.

S1: Vandana V, Karuna M, Vijayalakshmi P, Prasad R. A Simple Method to Enrich Phospholipid Content in Commercial Soybean Lecithin. Journal of the American Oil Chemists’ Society 2001;78:555-556.

S2: LOVERN JA, OLLEY J. The lipids of fish. II. The acetone-soluble lipids of the flesh of the haddock. The Biochemical journal 1953;54:128-37.

## Data handling

The acquired mass spectrometry (MS) data (scan range 33-400 m/z) were processed using the MS Workstation Software (Varian V6.9.3).

The retention index and the mass spectra of the analytes were compared to an internal database of 1800 known compounds. The peak area for each compound was calculated using the specific masses and is reported in “counts”. Peak areas for all metabolites were first normalized on the overall median total peak area of all samples. Secondly, the two extractions of each patient and mix sample were merged retaining the average of the two peak areas for each metabolite. Secondly, the ratio of the median area for each metabolite in the mix standards during a measurement batch as compared to its overall mean was calculated and the metabolite counts of the patient samples adjusted accordingly.

## Quantification

To transform the peak area counts obtained from the GC/MS into a quantitative estimate of compound amounts, a substance-specific response factor was be obtained from multiple SPME of a sample vial containing known amounts of the analyte. Such multiple SPME were performed on vials containing 75 ng of synthetic azelaic acid dimethylester (chiracon GmbH, Luckenwalde, Germany). Supplementary Figure 2A demonstrates the declining peak areas after thirteen SPME steps from the same vial. The decline of the peak area with each subsequent extraction follows the expected exponential curve (*r^2^*=0.994). The peak area of the last extraction amounts to less than 1 percent (Supplementary Table 2) of the first extraction. Therefore, the sum of the thirteen peak areas may be taken as the total peak area of 1.69×10^9^ counts corresponding to 75 ng (the total spiked amount) of the ester in the vial, yielding a specific response factor of 2.25×10^7^ counts/ng. Using this response factor, the amount of the analyte extracted in one step can be calculated by division of the peak area (Supplementary Table 2, column 2) by this response factor. The amount of analyte in the vial is be reduced by the extracted amount (Supplementary Table 2, columns 3 and 4). Further, the ratio of the peak area of the analyte and the amount in the vial was nearly constant, in the case of the dimethylester of azelainic acid about 1×10^7^ counts/ng (Supplementary Table 2, column 5). Thus, from a single extraction the amount (ng) in the vial may be estimated by division the peak area in counts by 1.0×10^7^ counts/ng (Supplementary Figure 2B).

## Statistics

Data processing and statistical analyses were using R ([www.r-project.org](http://www.r-project.org)) [R development core team]. For statistical analyses of compounds after normalisation, the decadic logarithm (log_10_) of the metabolite counts was used in order to achieve an approximate normal distribution for parametric analyses. The test variables (i.e. the log10 differences of metabolites) were tested for deviation from normality using the Komolgorov-Smirnov test using the R procedure ks.test() adopting a threshold of p<0.05. Principal components analyses were performed using the prcomp() function in R [Mardia et al]. Single metabolite analyses were performed t.test() and Wilcox.test(). Analysis of variance was performed using the aov() procedure.

R Development Core Team. R: A language and environment for statistical computing. Vienna, Austria: R Foundation for Statistical Computing; 2010.

Mardia KV, Kent JT, Bibby JM. Multivariate Analysis. London: Academic Press; 1979.

## Quality control and filtering of metabolites

As a first analysis step, a weighted control score reflecting the relative abundance of metabolites in the patient and mix samples in comparison to the control vials was calculated. For metabolites that were not detected in the control vials, an arbitrary control score of 1000 was assigned. Metabolites that were at least five times more abundant in the patient and mix samples (i.e. had a control score of greater 5) or were not present in the empty control vials in both the discovery and replication series were selected for further analysis. Further, metabolites with significant deviation of the pair-wise log_10_ differences from normality in the discovery series (i.e. p<0.05 in the Komolgorov-Smirnov test) were excluded. A total of 208 chemically defined substances matched these criteria and were thus available for further analysis of the effects of ES on liver tissue. A list of all analysed compounds is provided in Supplementary Table 1. Two compounds (FAME C12:0 and FAME C14:0) showed deviations of the log_10_ differences from normality (0.02 and 0.0004, respectively) in the replication set and are marked accordingly in Supplementary Table 1.
